# *catena*-Poly[[bis­(3-benzoyl­pyridine-κ*N*)zinc(II)]-di-μ-dicyanamido-κ^4^ *N* ^1^:*N* ^5^]

**DOI:** 10.1107/S1600536808019880

**Published:** 2008-07-05

**Authors:** Fei Yu, Zhong-Shu Li, Bai-Wang Sun

**Affiliations:** a Ordered Matter Science Research Center, College of Chemistry and Chemical Engineering, Southeast University, Nanjing 210096, People’s Republic of China

## Abstract

The title compound, [Zn(C_2_N_3_)_2_(C_12_H_9_NO)_2_]_*n*_, is a polymeric zinc(II) complex with the metal ion located on an inversion centre. The Zn^II^ ion is six-coordinated by two N atoms of two 3-benzoyl­pyridine ligands and four N atoms from four dicyanamide ligands, forming a slightly distorted octa­hedral configuration. In the crystal structure, neighboring Zn atoms are linked together by double dicyanamide bridges to form a polymeric zinc(II) complex.

## Related literature

For related literature, see: Armentano *et al.* (2006 ▶); Claramunt *et al.* (2000 ▶); Manson *et al.* (1998 ▶); Miller (2006 ▶).
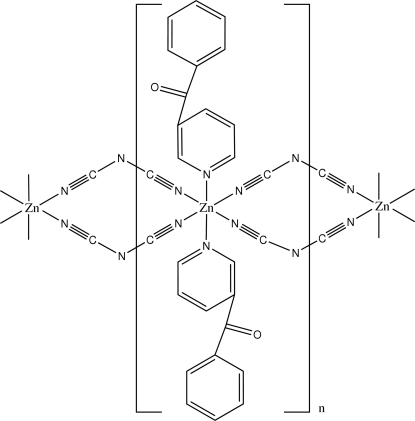


## Experimental

### 

#### Crystal data

[Zn(C_2_N_3_)_2_(C_12_H_9_NO)_2_]
*M* *_r_* = 563.89
Monoclinic, 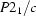

*a* = 6.463 (4) Å
*b* = 7.490 (4) Å
*c* = 26.300 (15) Å
β = 98.399 (16)°
*V* = 1259.5 (13) Å^3^
*Z* = 2
Mo *K*α radiation
μ = 1.02 mm^−1^
*T* = 293 (2) K
0.07 × 0.04 × 0.03 mm

#### Data collection

Rigaku Scxmini 1K CCD area-detector diffractometer
Absorption correction: multi-scan (*CrystalClear*; Rigaku, 2005 ▶) *T*_min_ = 0.867, *T*_max_ = 1.000 (expected range = 0.841–0.970)
12079 measured reflections
2880 independent reflections
2342 reflections with *I* > 2σ(*I*)
*R*_int_ = 0.045

#### Refinement

*R*[*F* ^2^ > 2σ(*F* ^2^)] = 0.050
*wR*(*F* ^2^) = 0.129
*S* = 1.09
2880 reflections
178 parameters
H-atom parameters constrained
Δρ_max_ = 0.60 e Å^−3^
Δρ_min_ = −0.56 e Å^−3^

### 

Data collection: *CrystalClear* (Rigaku, 2005 ▶); cell refinement: *CrystalClear*; data reduction: *CrystalClear* ; program(s) used to solve structure: *SHELXS97* (Sheldrick, 2008 ▶); program(s) used to refine structure: *SHELXL97* (Sheldrick, 2008 ▶); molecular graphics: *SHELXTL* (Sheldrick, 2008 ▶); software used to prepare material for publication: *SHELXTL*.

## Supplementary Material

Crystal structure: contains datablocks I, global. DOI: 10.1107/S1600536808019880/bq2086sup1.cif

Structure factors: contains datablocks I. DOI: 10.1107/S1600536808019880/bq2086Isup2.hkl

Additional supplementary materials:  crystallographic information; 3D view; checkCIF report

## Figures and Tables

**Table:** 

Zn1—N2	2.162 (3)
Zn1—N3	2.169 (2)
Zn1—N4	2.172 (3)

**Table:** 

N2—Zn1—N3	92.27 (11)
N2—Zn1—N4	90.79 (9)
N3—Zn1—N4	89.87 (9)

**Fig. 1.**
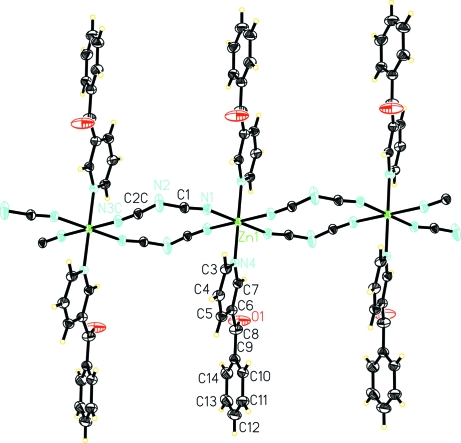
The molecular structure of the title compound with the atom-numbering scheme and all dydrogen atoms. Displacement ellipsoids are drawn at the 30% probability level.

**Fig. 2.**
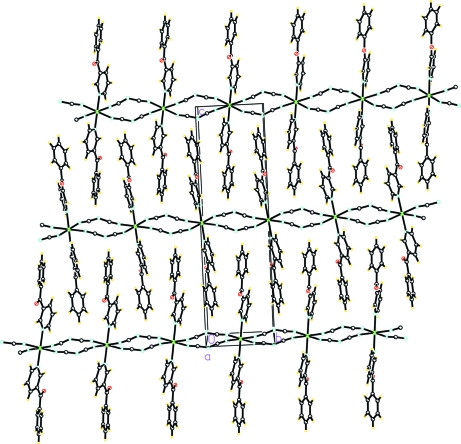
One-dimensional structure in the title compound. Displacement ellipsoids are drawn at the 30% probability level and all dydrogen atoms.

## supplementary crystallographic information

### Comment

The dicyanamide ligand has frequently been used to bridge polynuclear transition metal complexes in the study of multidimensional molecule-based magnetic materials and other areas. Many such compounds have been reported (Manson *et al.*, 1998; Claramunt *et al.*, 2000; Armentano *et al.*, 2006; Miller, 2006;). Here, we report the structure of the title Zn^II^ compound, (I). The structure of (I) is illustrated in Fig. 1, and bond distances and angles are given in Table 1. The Zn^II^ ion, which lies on the inversion centre, is in an octahedral geometry and is six-coordinated by six N atoms, from four dicyanamide ligands and two 3-benzoylpyridine ligands in a *trans* arrangement. The resulting coordination geometry is very close to that expected for an ideal octahedral complex. In the crystal structure, the Zn^II^ ions are bridged to form a one-dimensional chain by dicyanamide ligands, through single end-to-end coordination, the dicyanamide ligand acts as a bidentate bridging ligand by coordinating to adjacent Zn^II^ centres through its two terminal nitrile N atoms. No significant contacts are observed between adjacent chains in the crystal structure.

### Experimental

All chemicals used (reagent grade) were commercially available. 3-benzoylpyridine (18.3 mg, 0.1 mmol) was added slowly with stirring in aqueous solution (5 ml) of Zn(CH_3_COO)_2_.2H_2_O (21.9 mg, 0.1 mmol) and then sodium dicyanamide (17.8 mg, 0.2 mmol) in aqueous solution (5 ml) was added slowly. The resulting colorless solution was continuously stirred for about 30 min at room temperature and then filtered. The filtrate was slowly evaporated at room temperature over several days, and colorless needles crystals suitable for X-ray analysis were obtained.

### Refinement

Positional parameters of all H atoms were calculated geometrically.

### Crystal data

**Table:**

[Zn(C_2_N_3_)_2_(C_12_H_9_NO)_2_]	*F*_000_ = 576
*M**_r_* = 563.89	*D*_x_ = 1.487 Mg m^−^^3^
Monoclinic, *P*2_1_/*c*	Mo *K*α radiation λ = 0.71073 Å
Hall symbol: -P 2ybc	Cell parameters from 2637 reflections
*a* = 6.463 (4) Å	θ = 3.1–27.5º
*b* = 7.490 (4) Å	µ = 1.02 mm^−^^1^
*c* = 26.300 (15) Å	*T* = 293 (2) K
β = 98.399 (16)º	Block, colorless
*V* = 1259.5 (13) Å^3^	0.07 × 0.04 × 0.03 mm
*Z* = 2	

### Data collection

**Table:**

Rigaku Scxmini 1K CCD area-detector diffractometer	2880 independent reflections
Radiation source: fine-focus sealed tube	2342 reflections with *I* > 2σ(*I*)
Monochromator: graphite	*R*_int_ = 0.045
Detector resolution: 8.192 pixels mm^-1^	θ_max_ = 27.5º
*T* = 293(2) K	θ_min_ = 3.1º
thin–slice ω scans	*h* = −8→8
Absorption correction: Multi-scan(CrystalClear; Rigaku, 2005)	*k* = −9→9
*T*_min_ = 0.867, *T*_max_ = 1.000	*l* = −34→33
12079 measured reflections	

### Refinement

**Table:**

Refinement on *F*^2^	Secondary atom site location: difference Fourier map
Least-squares matrix: full	Hydrogen site location: inferred from neighbouring sites
*R*[*F*^2^ > 2σ(*F*^2^)] = 0.050	H-atom parameters constrained
*wR*(*F*^2^) = 0.129	*w* = 1/[σ^2^(*F*_o_^2^) + (0.0563*P*)^2^ + 0.6329*P*] where *P* = (*F*_o_^2^ + 2*F*_c_^2^)/3
*S* = 1.09	(Δ/σ)_max_ < 0.001
2880 reflections	Δρ_max_ = 0.60 e Å^−^^3^
178 parameters	Δρ_min_ = −0.56 e Å^−^^3^
Primary atom site location: structure-invariant direct methods	Extinction correction: none

### Special details

**Table:**

Geometry. All e.s.d.'s (except the e.s.d. in the dihedral angle between two l.s. planes) are estimated using the full covariance matrix. The cell e.s.d.'s are taken into account individually in the estimation of e.s.d.'s in distances, angles and torsion angles; correlations between e.s.d.'s in cell parameters are only used when they are defined by crystal symmetry. An approximate (isotropic) treatment of cell e.s.d.'s is used for estimating e.s.d.'s involving l.s. planes.
Refinement. Refinement of *F*^2^ against ALL reflections. The weighted *R*-factor *wR* and goodness of fit *S* are based on *F*^2^, conventional *R*-factors *R* are based on *F*, with *F* set to zero for negative *F*^2^. The threshold expression of *F*^2^ > σ(*F*^2^) is used only for calculating *R*-factors(gt) *etc*. and is not relevant to the choice of reflections for refinement. *R*-factors based on *F*^2^ are statistically about twice as large as those based on *F*, and *R*- factors based on ALL data will be even larger.

### Fractional atomic coordinates and isotropic or equivalent isotropic displacement parameters (Å^2^)

**Table:**

	*x*	*y*	*z*	*U*_iso_*/*U*_eq_	
Zn1	0.5000	−0.5000	0.0000	0.03523 (16)	
O1	0.1648 (4)	−0.5269 (5)	−0.20379 (11)	0.1080 (15)	
N2	0.7192 (4)	−0.2827 (3)	0.01736 (10)	0.0467 (6)	
N3	0.7294 (4)	−0.6966 (3)	0.03187 (9)	0.0441 (6)	
N4	0.5973 (4)	−0.5346 (3)	−0.07500 (9)	0.0374 (5)	
N5	0.8571 (7)	−0.9980 (3)	0.05024 (17)	0.0911 (15)	
C1	0.7821 (4)	−0.8411 (4)	0.03808 (11)	0.0405 (6)	
C2	0.7750 (4)	−0.1441 (4)	0.03062 (11)	0.0411 (6)	
C4	0.7925 (4)	−0.5898 (4)	−0.07850 (11)	0.0442 (7)	
H4A	0.8899	−0.5924	−0.0487	0.053*	
C5	0.8557 (5)	−0.6427 (4)	−0.12406 (11)	0.0488 (7)	
H5C	0.9914	−0.6831	−0.1247	0.059*	
C6	0.7130 (5)	−0.6347 (4)	−0.16892 (11)	0.0465 (7)	
H6A	0.7504	−0.6728	−0.2000	0.056*	
C7	0.5141 (5)	−0.5690 (4)	−0.16669 (11)	0.0435 (6)	
C8	0.4633 (4)	−0.5223 (3)	−0.11864 (11)	0.0395 (6)	
H8A	0.3290	−0.4805	−0.1169	0.047*	
C9	0.3436 (5)	−0.5503 (5)	−0.21175 (12)	0.0568 (8)	
C10	0.3845 (5)	−0.5665 (4)	−0.26610 (11)	0.0491 (7)	
C11	0.5699 (6)	−0.5147 (4)	−0.28267 (13)	0.0553 (8)	
H11A	0.6808	−0.4735	−0.2591	0.066*	
C12	0.2197 (6)	−0.6285 (5)	−0.30199 (13)	0.0635 (9)	
H12A	0.0948	−0.6634	−0.2913	0.076*	
C13	0.2419 (8)	−0.6382 (5)	−0.35361 (14)	0.0783 (13)	
H13A	0.1321	−0.6804	−0.3774	0.094*	
C14	0.4252 (8)	−0.5857 (6)	−0.36965 (15)	0.0811 (14)	
H14A	0.4386	−0.5915	−0.4043	0.097*	
C15	0.5896 (8)	−0.5245 (5)	−0.33469 (14)	0.0707 (12)	
H15A	0.7137	−0.4898	−0.3458	0.085*	

### Atomic displacement parameters (Å^2^)

**Table:**

	*U*^11^	*U*^22^	*U*^33^	*U*^12^	*U*^13^	*U*^23^
Zn1	0.0407 (3)	0.0283 (2)	0.0361 (3)	0.00125 (17)	0.00358 (18)	−0.00050 (17)
O1	0.0444 (14)	0.232 (5)	0.0468 (15)	0.0126 (19)	0.0035 (12)	−0.0020 (19)
N2	0.0456 (13)	0.0353 (13)	0.0569 (15)	−0.0031 (10)	0.0002 (11)	−0.0015 (11)
N3	0.0486 (14)	0.0348 (13)	0.0479 (14)	0.0061 (10)	0.0033 (11)	0.0023 (10)
N4	0.0400 (12)	0.0358 (12)	0.0364 (11)	0.0012 (9)	0.0055 (9)	0.0000 (9)
N5	0.097 (3)	0.0326 (15)	0.121 (3)	0.0078 (15)	−0.060 (2)	−0.0090 (15)
C1	0.0391 (14)	0.0354 (15)	0.0438 (15)	0.0003 (11)	−0.0044 (12)	−0.0041 (11)
C2	0.0414 (15)	0.0348 (15)	0.0444 (15)	0.0057 (12)	−0.0024 (12)	0.0006 (12)
C4	0.0394 (14)	0.0520 (18)	0.0407 (15)	0.0017 (13)	0.0047 (12)	0.0034 (13)
C5	0.0421 (15)	0.0588 (19)	0.0459 (16)	0.0102 (14)	0.0078 (13)	0.0037 (14)
C6	0.0481 (16)	0.0505 (17)	0.0432 (15)	0.0007 (13)	0.0142 (13)	−0.0029 (13)
C7	0.0442 (15)	0.0478 (16)	0.0387 (15)	−0.0025 (13)	0.0065 (12)	−0.0015 (12)
C8	0.0390 (14)	0.0392 (15)	0.0402 (14)	0.0013 (11)	0.0052 (11)	0.0001 (11)
C9	0.0495 (18)	0.079 (2)	0.0413 (17)	0.0025 (17)	0.0050 (14)	0.0018 (16)
C10	0.0579 (19)	0.0500 (17)	0.0378 (15)	0.0099 (15)	0.0017 (13)	−0.0014 (13)
C11	0.071 (2)	0.0519 (19)	0.0445 (17)	0.0105 (16)	0.0125 (16)	0.0005 (14)
C12	0.068 (2)	0.066 (2)	0.0512 (19)	0.0146 (18)	−0.0106 (16)	−0.0011 (16)
C13	0.105 (3)	0.072 (3)	0.048 (2)	0.034 (2)	−0.021 (2)	−0.0112 (18)
C14	0.119 (4)	0.081 (3)	0.043 (2)	0.044 (3)	0.011 (2)	0.0005 (19)
C15	0.097 (3)	0.070 (3)	0.049 (2)	0.028 (2)	0.025 (2)	0.0075 (17)

### Geometric parameters (Å, °)

**Table:**

Zn1—N2	2.162 (3)	C6—C7	1.385 (4)
Zn1—N2^i^	2.162 (3)	C6—H6A	0.9300
Zn1—N3^i^	2.169 (2)	C7—C8	1.396 (4)
Zn1—N3	2.169 (2)	C7—C9	1.502 (4)
Zn1—N4	2.172 (3)	C8—H8A	0.9300
Zn1—N4^i^	2.172 (3)	C9—C10	1.496 (4)
O1—C9	1.217 (4)	C10—C11	1.389 (5)
N2—C2	1.137 (4)	C10—C12	1.395 (5)
N3—C1	1.139 (4)	C11—C15	1.395 (5)
N4—C8	1.337 (4)	C11—H11A	0.9300
N4—C4	1.343 (4)	C12—C13	1.388 (5)
N5—C2^ii^	1.290 (4)	C12—H12A	0.9300
N5—C1	1.293 (4)	C13—C14	1.372 (6)
C2—N5^iii^	1.290 (4)	C13—H13A	0.9300
C4—C5	1.379 (4)	C14—C15	1.378 (6)
C4—H4A	0.9300	C14—H14A	0.9300
C5—C6	1.389 (4)	C15—H15A	0.9300
C5—H5C	0.9300		
			
N2—Zn1—N2^i^	180.00 (9)	C7—C6—H6A	120.5
N2—Zn1—N3^i^	87.73 (11)	C5—C6—H6A	120.5
N2^i^—Zn1—N3^i^	92.27 (11)	C6—C7—C8	118.0 (3)
N2—Zn1—N3	92.27 (11)	C6—C7—C9	125.3 (3)
N2^i^—Zn1—N3	87.73 (11)	C8—C7—C9	116.6 (3)
N3^i^—Zn1—N3	180.0	N4—C8—C7	123.5 (3)
N2—Zn1—N4	90.79 (9)	N4—C8—H8A	118.3
N2^i^—Zn1—N4	89.21 (9)	C7—C8—H8A	118.3
N3^i^—Zn1—N4	90.13 (9)	O1—C9—C10	118.7 (3)
N3—Zn1—N4	89.87 (9)	O1—C9—C7	118.9 (3)
N2—Zn1—N4^i^	89.21 (9)	C10—C9—C7	122.3 (3)
N2^i^—Zn1—N4^i^	90.79 (9)	C11—C10—C12	119.3 (3)
N3^i^—Zn1—N4^i^	89.87 (9)	C11—C10—C9	123.9 (3)
N3—Zn1—N4^i^	90.13 (9)	C12—C10—C9	116.7 (3)
N4—Zn1—N4^i^	180.00 (4)	C10—C11—C15	120.0 (4)
C2—N2—Zn1	157.0 (2)	C10—C11—H11A	120.0
C1—N3—Zn1	150.9 (2)	C15—C11—H11A	120.0
C8—N4—C4	117.4 (2)	C13—C12—C10	120.1 (4)
C8—N4—Zn1	122.30 (19)	C13—C12—H12A	120.0
C4—N4—Zn1	119.90 (18)	C10—C12—H12A	120.0
C2^ii^—N5—C1	123.7 (3)	C14—C13—C12	120.2 (4)
N3—C1—N5	172.8 (3)	C14—C13—H13A	119.9
N2—C2—N5^iii^	172.0 (3)	C12—C13—H13A	119.9
N4—C4—C5	123.2 (3)	C13—C14—C15	120.4 (4)
N4—C4—H4A	118.4	C13—C14—H14A	119.8
C5—C4—H4A	118.4	C15—C14—H14A	119.8
C4—C5—C6	118.8 (3)	C14—C15—C11	120.0 (4)
C4—C5—H5C	120.6	C14—C15—H15A	120.0
C6—C5—H5C	120.6	C11—C15—H15A	120.0
C7—C6—C5	119.0 (3)		
			
N3^i^—Zn1—N2—C2	40.5 (6)	C4—N4—C8—C7	2.1 (4)
N3—Zn1—N2—C2	−139.5 (6)	Zn1—N4—C8—C7	−170.4 (2)
N4—Zn1—N2—C2	130.6 (6)	C6—C7—C8—N4	1.3 (4)
N4^i^—Zn1—N2—C2	−49.4 (6)	C9—C7—C8—N4	178.9 (3)
N2—Zn1—N3—C1	−168.8 (5)	C6—C7—C9—O1	164.8 (4)
N2^i^—Zn1—N3—C1	11.2 (5)	C8—C7—C9—O1	−12.6 (5)
N4—Zn1—N3—C1	−78.0 (5)	C6—C7—C9—C10	−12.8 (5)
N4^i^—Zn1—N3—C1	102.0 (5)	C8—C7—C9—C10	169.8 (3)
N2^i^—Zn1—N4—C8	58.2 (2)	O1—C9—C10—C11	150.0 (4)
N3^i^—Zn1—N4—C8	−34.0 (2)	C7—C9—C10—C11	−32.4 (5)
N3—Zn1—N4—C8	146.0 (2)	O1—C9—C10—C12	−26.4 (5)
N2—Zn1—N4—C4	65.9 (2)	C7—C9—C10—C12	151.2 (3)
N2^i^—Zn1—N4—C4	−114.1 (2)	C12—C10—C11—C15	0.2 (5)
N3^i^—Zn1—N4—C4	153.6 (2)	C9—C10—C11—C15	−176.1 (3)
N3—Zn1—N4—C4	−26.4 (2)	C11—C10—C12—C13	0.1 (5)
C8—N4—C4—C5	−3.7 (4)	C9—C10—C12—C13	176.6 (3)
Zn1—N4—C4—C5	169.0 (2)	C10—C12—C13—C14	−0.5 (6)
N4—C4—C5—C6	1.7 (5)	C12—C13—C14—C15	0.6 (6)
C4—C5—C6—C7	1.8 (5)	C13—C14—C15—C11	−0.4 (6)
C5—C6—C7—C8	−3.2 (5)	C10—C11—C15—C14	0.0 (5)
C5—C6—C7—C9	179.4 (3)		

Symmetry codes: (i) −*x*+1, −*y*−1, −*z*; (ii) *x*, *y*−1, *z*; (iii) *x*, *y*+1, *z*.
